# Factors affecting medical students’ interests in working in rural areas in North India—A qualitative inquiry

**DOI:** 10.1371/journal.pone.0210251

**Published:** 2019-01-10

**Authors:** Sonu Goel, Federica Angeli, Nonita Dhirar, Garima Sangwan, Kanchan Thakur, Dirk Ruwaard

**Affiliations:** 1 Department of Community Medicine and School of Public Health, Post Graduate Institute of Medical Education and Research, Chandigarh, India; 2 Department of Health Services Research, Care and Public Health Research Institute (CAPHRI), Faculty of Health, Medicine and Life Sciences, Maastricht University, Maastricht, The Netherlands; University of Toronto, Rotman School, CANADA

## Abstract

**Background and Objective:**

The shortage of doctors, especially in rural areas, is a major concern in India, which in turn affects the effective delivery of health care services. To support new policies able to address this issue, a study was conducted to determine the discouraging and encouraging factors affecting medical students’ interests towards working in rural areas.

**Methods:**

This cross-sectional, descriptive qualitative study has been conducted in three states of North India. It comprised six focus group discussions, each consisting of 10–20 medical students of six government medical colleges. The verbatim and thematic codes have been transcribed by using a ‘categorical aggregation approach’. The discussions were thematically analyzed.

**Results:**

Ninety medical students participated in the study. The discouraging factors were grouped under two broad themes namely *unchallenging professional environment* (poor accommodation facilities and lack of necessary infrastructure; lack of drug and equipment supplies; inadequate human resource support; lesser travel and research opportunities) and *gap between financial rewards and social disadvantages* (lower salary and incentives, social isolation, political interference, lack of security). Similarly, the encouraging factors were congregated under three main themes namely *willingness to give back to disadvantaged communities* (desire to serve poor, underprivileged and home community), *broader clinical exposure* (preferential admission in post-graduation after working more than 2–3 years in rural areas) and *higher status and respect* (achieving higher social status).

**Conclusions:**

This qualitative study highlights key factors affecting medical students’ interest to work in rural areas. A substantial similarity was noted between the factors which emerge from the current study and those documented in other countries. These findings will help policymakers and medical educators to design and implement a comprehensive human resource strategy that shall target specific factors to encourage medical students to choose job positions in rural areas.

## Introduction

There is a shortage of doctors globally, especially in rural and remote areas, which hinders the progress towards improving the health of people in these areas [1–2]. This global challenge has been accorded high priority by the World Health Organization (WHO) [3]. Against the optimal threshold of 4.45 skilled health professionals including one doctor per 1,000 population, the estimated shortage is17.4 million experienced professionals and 2.6 million doctors globally, with the greatest shortage in the high disease burden countries of South East Asia and African regions [4]. There is a need to increase the health workforce for achieving essential health services in both low income and upper-middle-income countries [5]. Current data document that more than44% of WHO member states have fallen short of the WHO standard of one physician per 1,000 populations [6].

India, with a population of over 1 billion, is placed in the lowest category of human resources for health (HRH) indicators [7]. A large number of trained doctors leave India for residency training and to work abroad, while the remaining works in urban areas. This pattern has resulted in an uneven distribution of doctors in India [8]. Rural health statistics-India 2016 reports a shortfall of 11.6% of medical officers at the primary health care level and 81.2% of medical specialists at community health centers [9]. The gap between the numbers of doctors working in urban areas as compared to rural areas is widening, which is endangering the functioning and sustainability of the Indian health care system [10–11].

A few studies conducted in developed countries, such as the UK, Australia, New Zealand, Croatia, and Hungary, had assessed the perception and attitudes of medical students towards rural settings. They observed that social isolation, poor housing support, poor equipment supply, need for career development and inadequate incentives were the primary deterrents for doctors towards working in rural areas [12–16]. The studies from developing countries like Bangladesh, India, Malawi, and Ghana had also shown similar results citing factors like accommodation, infrastructure, poor resources and financial motives [17–21] as major deterrents by medical students to work in rural areas. A mixture of factors was cited by elements of other studies from both developed and developing countries [22–25].

Various strategies have been implemented in the health sector to recruit and retain health professionals in rural and remote areas [12,16,26]. These measures have attempted to address not only financial incentives, but also non-financial incentives like living conditions, education opportunities for employees’ children, and future career prospects [12,16,21]. Numerous efforts were also made by the Government of India to promote postings in rural areas, such as preferential admission in post-graduation after working for a fixed time period in rural areas [18,19], compulsory rural field experience during medical studies and rural service after medical graduation [18], or rural allowances [18–19]. However, they have tasted only sporadic success, primarily because of the implementation of isolated strategies rather than bundled interventions [8,10–11,18–19]. Moreover, relatively little research has been done in India, on understanding the perceptions of medical students about the factors that can promote rural practice [10–12].

Medical education in India consists of a five and a half years course of medical teaching in a tertiary health care institution which includes one year of compulsory internship. The medical study focuses on basic pre-clinical subjects for the first two and a half years andclinical subjects for the next two years. The students simultaneously obtain hands-on-training in the wards and outpatient departments, where they interact with real patients. The four and half years of medical study are followed by one year of internship wherein the medical students (now called interns) undergo compulsory rotational postings in various clinical disciplines. Postings can be conducted in the medical college hospital or any approved hospital facility(also known as Teaching Hospital)in order to obtain practical experience under supervision of senior doctors. Apart from being posted in other clinical disciplines, the interns are compulsorily posted for three months in the community where they not only practice skills in patient care accumulated during earlier postings but also learn comprehensive care including preventive, promotive, curative and rehabilitative services. The interns do not have a full license to practice medicine unsupervised by the Medical Council of India and are being paid a meager stipend to sustain their expenditures. After the period of internship, the interns are expected to acquire competencies to deal effectively with an individual and the community in the context of primary health care during their posting in government-funded primary or secondary care hospitals. The primary care units are essentially single-physician clinics usually with facilities for minor surgeries, whereas, secondary care centers are referral units with advanced specialized facilities like obstetric and newborn care, major surgical facilities and blood storage capacities at all hours every day.

In countries like India, where government funds bear the cost of medical education for the majority of its medical students, reliable measures for retaining health professionals in rural areas is of prime concern. The existing research demonstrates that it is possible for medical schools to combat the dearth of doctors in rural areas by selecting medical students from a rural background and also providing medical education in a rural setting [12,16,18]. However, there is insufficient evidence from India in support of these ideas as fewer studies have been conducted [18,19].

Therefore, understanding the perception of medical students about the challenges of rural areas could predict their subsequent practice location and help stimulate their choice to work or stay in rural areas. The present study aims at exploring medical students’ interest in working in rural settings in India by examining the discouraging and encouraging factors.

## Material and methods

### Study design

This research used qualitative methods of inquiry in order to explore the discouraging and encouraging factors affecting medical students’ interests in working in rural areas, along with understanding the context and mechanisms behind their interests. The entire process of conducting the FGD was based on standard literature on qualitative research [27].

### Study setting

The study has been conducted in three northern states of India (Himachal Pradesh, Haryana, and Punjab) which have a combined population of around 60 million [28]. These three states have a total of six government medical colleges. The states are almost equal in area, size and have poor health indicators as compared to the southern part of India. Many areas in these northern states are predominantly ‘rural’ and categorized by low population density with poor facilities for doctors, hence face difficulty in recruiting and retaining them.

### Study population

The current qualitative research is a part of broader mixed method study which included 636 medical students (including interns) of six government medical colleges of three states viz. Himachal Pradesh (HP), Punjab and Haryana located in Northern India. Of the 636 medical students, 297 (53.3%) were males, and 339 (46.7%) were females. The average age for males was 22.4 years (SD = 2.03) and for females 22.10 years (SD = 1.53). Most (97.8%) were unmarried, 81% of students had completed their premedical studies in urban areas, and 40.7% belonged to rural family background. Final year students of Bachelor of Medicine, Bachelor of Surgery (MBBS) and interns were selected as they are near to complete their medical education and have to decide their posting in rural or urban areas.

After obtaining quantitative data, focus group discussions (FGDs) were conducted among few of them. A variable number (between 10 and 20) of students from each medical college was enrolled in an FGD, thus totaling to 90 students from 6 FGDs. The students who were very vocal had experienced a clinical environment and were thought to provide the best information in the form of positive or negative comments (‘information rich’ cases) were purposively included in the FGD, to guarantee sample heterogeneity. The selection process ensured the homogeneity of participants in terms of year of medical study and at least one other characteristic among gender, medical school admission process or rural interest. These selection criteria were applied because they can contribute to the student’s attitude towards rural practice [29, 30].Representativeness of the college population was ensured by including all the government medical colleges in the three states. Representativeness on other dimensions such as age, gender, ethical/religious background, and geographical origin was also assured. [27,31] When the researchers observed that no new themes emerged in the last FGDs, they deemed that saturation had been achieved and that no additional FGDs were necessary.

### Study tool

Focus group guidelines with open-ended questions were developed based on the existing literature [11,12,16,18,29]. FGDs were conductedto explore information about the topic of interest using structured discussions. The trained facilitators used a pre-prepared topic list of questions on discouraging and encouraging factors related to working in rural areas. Further, students were asked to recommend possible interventions that may be effective to improve attraction and retention of doctors in rural areas. The questions used for the FGDs are shown in Table 1.

**Table 1:** Questions asked during focus group discussions to identify encouraging and discouraging factors for working in rural areas.

How many of you would like to serve in rural areas after graduation? What, according to you are the motivational factors of working in a rural setup? What, would you say, are the possible challenges faced while working in a rural setting? What facilities have been provided by the government to retain doctors in rural areas? What provisions should be made available by the government to encourage doctors to work in rural areas?

### Data collection

A prior written informed consent was taken from the participants. The participants were ensured about their anonymity and confidentiality while reporting the results. The necessary permissions were also obtained from Principals of medical colleges in each state.

The research team consisted of two research assistants (Masters of Public Health with two years of research experience) and one research associate (Ph.D.), who were well versed with local languages and trained in qualitative research methods. The research assistants conducted the FGDs, one being the moderator and the other the facilitator. The FGDs were conducted in the native local language, at a suitable time and place convenient to the participants. The purpose of the study has been explained to the participants, to allow for aninformed participation choice. After an ‘icebreaker’ question, ‘discussion starter’ questions were floated among the participants for generating interest in the topic, facilitating discussion and deterring ‘group think’. After that, open-ended questions were asked to obtain the participants’ perspective on the problem. Each person was asked to provide a final summary statement before concluding the session. After that, the research associate concluded the discussion by summarizing the key statements raised during the FGD to ensure participants’ validation.

### Data analysis

The two research assistants took notes of the focus group session and independently examined and coded the transcripts for reduction of bias and interpretative credibility. After that, categories were developed representing themes in the subject area by combining similar codes (inductive approach). The consensus was obtained by further discussion of the final data with the research associate, principal investigator and subject experts (academicians and researchers (n = 6), program managers (n = 3) and policymakers (n = 3)). They also examined the transcripts separately to determine whether any new issues or themes had emerged, ensuring the completeness of ideas and reliability of the data. The verbatim and thematic codes were transcribed by using a ‘categorical aggregation approach’ [32,33]. The results of both medical students, as well as interns, were grouped during analysis since both were in the phase of decision-making regarding choosing to work in rural areas. The findings were reportedin accordance with the Qualitative Research reporting guidelines COREQ (Consolidated criteria for reporting qualitative research)[34].

This study has been approved by the Institute Ethical Committee (PGI/IEC/2012/810-I P- 154) of Post Graduate Institute of Medical Education and Research, Chandigarh, India.

## Results

Ninety students participated in the study with males (n = 51, 56.7%) outnumbering females (n = 39, 43.3%). The age range was 20–27 years (mean age 22.4 years, SD 2.03) and all were unmarried. Nearly one-third of the study subjects were from the state of Himachal Pradesh (n = 33; 36.7%), and quite similar numbers belonged to Punjab (n = 30; 33.3%) and Haryana (n = 27; 30%). There were almost equal numbers of final year medical students (n = 46, 51.1%) and interns (n = 44, 48.9%). Around one third (n = 32, 36%) belonged to the rural background while one fifth (n = 15, 20%) underwent premedical studies (i.e., prerequisite educational track during secondary schooling for obtaining admission in medical school) from rural areas. (Table 2)

**Table 2:** Descriptive statistics of the sample (n = 90).

	FGD 1 (n = 20) *	FGD 2 (n = 15)	FGD 3 (n = 10)	FGD 4 (n = 15)	FGD 5 (n = 10)	FGD 6 (n = 20)
Age group (in years)	22–27 years	22–27 years	22–27 years	22–27 years	22–27 years	22–27 years
Gender	Males = 10 Females = 10	Males = 8 Females = 7	Males = 6 Females = 4	Males = 9 Females = 6	Males = 8 Females = 2	Males = 10 Females = 10
Final year intern ratio	7:13	8:7	4:6	8:7	6:4	13:7
Geographical origin wise distribution	Himachal Pradesh = 15 Punjab = 2 Haryana = 3	Himachal Pradesh = 13 Punjab = 1 Haryana = 1	Himachal Pradesh = 1 Punjab = 8 Haryana = 1	Himachal Pradesh = 2 Punjab = 12 Haryana = 1	Himachal Pradesh = 1 Punjab = 3 Haryana = 6	Himachal Pradesh = 1 Punjab = 4 Haryana = 15
Rural background	7	5	4	6	5	5
Underwent premedical studies from rural areas	3	2	1	4	2	3

*figure in parenthesis denotes the number of medical students

The analysis revealed that the level of medical students’ interest in rural areas could be structured broadly into discouraging and encouraging factors. The discouraging factors were further grouped under two themes- *unchallenging professional environment* and *gap between financial rewards and social disadvantages*; whereas encouraging factors were grouped under three themes- *willingness to give back to disadvantaged communities*, *broader clinical exposure*, and *higher status and respect*. All students mentioned both discouraging and encouraging factors but stressed more on discouraging factors as compared to encouraging factors.

### Discouraging factors

#### Theme-1: Unchallenging professional environment

The students cited unavailability of cutting-edge technologies, inadequate human resource support, fewer opportunities to travel abroad and fewer research options as barriers to work in rural areas. Moreover, poor accommodation facilities along with lack of basic amenities (like inadequate hospital infrastructure, poor drug supply and lack of equipment) demotivate them to work in rural settings. The following quotes express the students’ opinions:

- ‘We can’t cater to diagnostic and curative needs of all the patients due to lack of modern equipment.’
- ‘There is no equipment, no medication, and no challenging work in rural areas.’
- ‘There is no staff in Primary Health Centers (PHCs), no helpers to assist us. How can a doctor work without human resource?’
- ‘The basic facilities, like electricity, proper infrastructure, accommodation, medicines, etc. are lacking. It is very difficult to work in such scenario.’

Some respondents expressed that they lacked the competence to serve in rural areas, as they were not adequately taught rural medicine and primary care during their medical education. They strongly felt a need to incorporate rural healthcare in the curriculum of medical education as depicted by their statements:

- ‘We are not confident to treat illnesses in a rural area as we are not taught rural medicine in our syllabus.’
- ‘There are atypical clinical cases in rural areas to which we have not been exposed. We lack knowledge for their effective management.’

The students believed that improved hospital infrastructure and supplies, provision of basic amenities in hospitals (for example, water supply, sanitation, electricity, accommodation), availability of communication facilities (roads, phones, internet) and preferential opportunity to go abroad for updating skills would incite them to work in rural areas. Some statements:

- ‘We want state-of-art technology, access to the latest medicines and equipment in rural setups so that our skills are not wasted.’
- ‘After five years of rural service, a doctor should be given an opportunity to visit abroad to learn about newer technologies.’

#### Theme-2: Gap between financial rewards and social disadvantages

The medical students felt that they would probably be isolated and left out in the remote and rural settings. Students have a general feeling that the salary package offered in rural areas does not fairly compensate for the benefits of working in urban areas. They wished that the salary package for doctors working in a rural area should be significantly more than the doctors serving in urban settings. They expressed their feelings as:

- ‘The salary and other emoluments should be significantly more for rural areas as compared to urban.’
- ‘The students who will be graduating by paying hefty admission fees will tend to work in the private hospitals in urban areas to make more money to compensate for hefty fees.’

Isolation from families, loneliness, boredom, and lack of work opportunities for a spouse were other factors, which repel medical students from working in rural areas. Political interference in the form of frequent transfers of doctors from one place to another, the insensitive attitude of the community leaders towards them, incidents of violence and lack of security also discourage them towards working in the rural area. The students mentioned the problems as follows:

- ‘We don’t want to live away from our families.’
- ‘Work options for spouse and schooling for children in rural areas are not as good as in urban areas.’
- ‘We have heard that few influential people threaten doctors to see their patients on priority, which compromises their security in an isolated rural setting.’
- ‘There should be less political interference and more security for doctors working in rural areas.’

### Encouraging factors

The most common encouraging factors for medical students’ towards working in rural and remote areas after completing graduation could be categorized under three major themes, as described below.

#### Theme-1: Willingness to give back to disadvantaged communities

The students had a desire to serve the poor and underprivileged communities who cannot afford quality medical services. Further, they feel responsible and privileged towards their family and community who have supported them during their studies. The respondents were also aware of the real unmet needs in rural areas. Their words describing the desire to give back to their people are as follows:

- ‘My parents have shown me the path and have supported me throughout my studies.’
- ‘I want to pay the debt to the community where I was born and studied by serving them.’

#### Theme-2: Broader clinical exposure

The medical students mentioned that they would gain better clinical experience while practicing on the diverse group of patients in rural areas, which would be unusual in urbanized settings. Also, they had the edge over their counterparts in getting preference for admission to post-graduation courses after a few years of rural service. The statements that summarise their perceptions are as below:

- ‘We would get more exposure to various types of cases in the rural areas.’
- ‘We want to get admission in post-graduation, so prefer to serve in rural areas.’

#### Theme-3: Higher status and respect

The medical students also perceived that they would get more respect in society if they practice in the rural community. They also opined that the people in rural areas are humble and appreciative of the medical profession. The students quoted their feelings as follows:

- ‘People in rural areas are more thankful unlike in urban areas.’
- ‘Our motivation increases when the community shows respect and gratitude for our hard work.’

## Discussion

This is the first qualitative study from the northern part of India, which explored the factors influencing medical students’ interest to work in rural areas. The earlier studies have evaluated isolated governmental effortsto retainhealth workforce in rural areas, such asfinancial incentives, compulsory rural field experience, and preferential admission in post-graduation [10–12].Little research on understanding the perceptions of medical students about the factors that can promote rural practice has been conducted, which could serve as good predictors for their subsequent practice location in rural areas. The themes generated out of the present exploratory qualitative study are in confirmation with the themes emanated from the quantitative development and validation study by Goel et al., hence strengthening the findings [35]. Further, the findings of the present study can be mirrored to the Maslow’s Hierarchy of needs theory for the better understanding of the issue of motivation of medical students towards working in rural areas [36].

**Fig 1:**
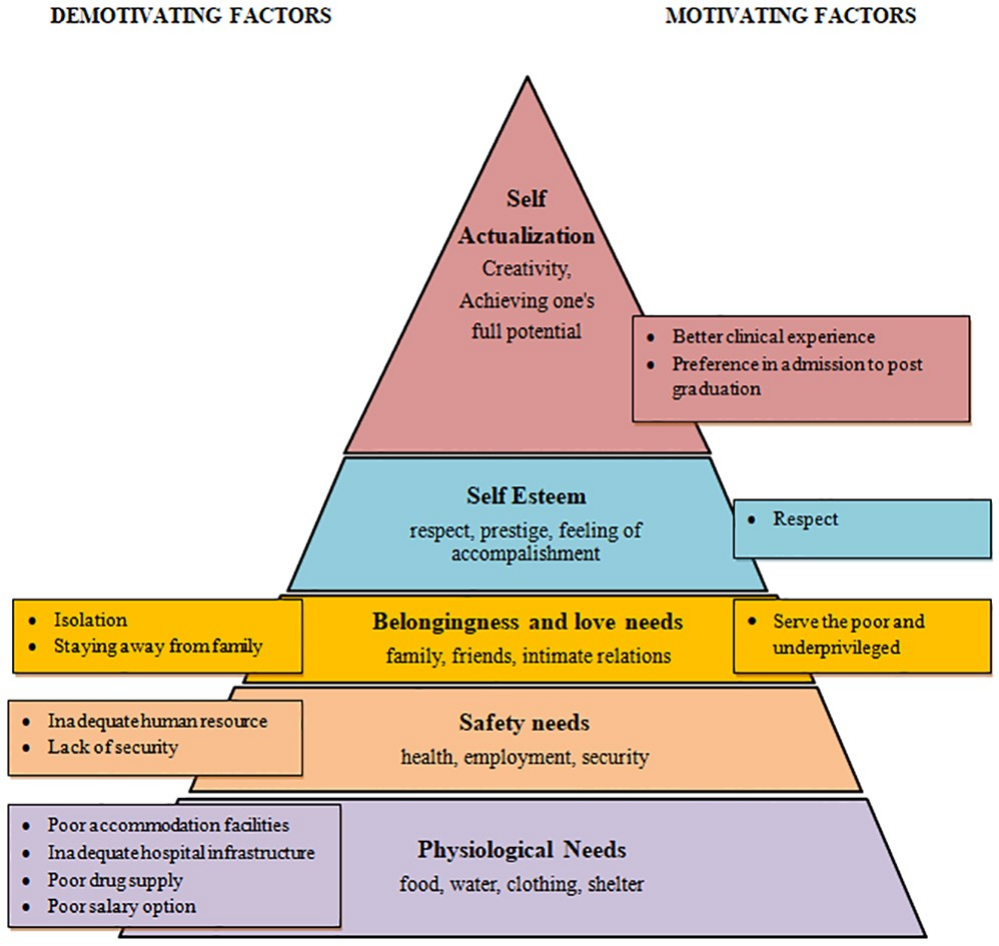
Factors motivating medical students to work in rural areas as depicted by Maslow’s Hierarchy of needs pyramid.

The five-stage “Maslow’s Hierarchy of Needs”behavioral model of motivation places from below upwards; physiological, safety, belongingness, self-esteem; and self-actualization needs (Fig 1). The theory states that the motivation of an individual undergoes a transition to higher segments (self-esteem, self-actualization) after fulfillment of needs in the lower segments (physiological, safety/security) of the pyramid. In the present study also, it was observed that the discouraging factors were the ones that lie in the lower segments of the pyramid and were stronger influencers of motivation as compared to encouraging factors. In low and middle-income countries, the students fall in the lower segments of the Maslow’s pyramid as they were still struggling to receive basic health care facilities and employment opportunities. Hence their major motivating factors were primarily humanitarian (like willingness to give back to disadvantaged communities), which is fulfilled, could lead to higher-level needs for motivation. Thus, Maslow’s Hierarchy of needs theory helps us to understand the issue of motivation of medical students towards working in rural areas.

The resultsof this researchare in linewith other studies that have identified *unchallenging professional environment* like hospital infrastructure [15,18,19,37,38,39], poor drug and equipment supply [15,19,38,39], lack of human resources [37–40], poor transport services [37,38,40], inadequate housing/accommodation [37–42] as the discouraging factors affecting the choice of medical students to work in rural settings. It is hardly surprising that doctors who were used to working with the best equipment in medical colleges lack the willingness to work in rural hospitals where these components are lacking. The medical graduates also consider it essential to treat patients to the best of their ability, which they perceived as less possible in rural hospitals. The students’ perception about the unavailability of cutting edge technology as one of the key discouraging factors highlights the faulty medical education of India which overemphasizes specialized care over basic medical services in rural areas. Thus, a curriculum based on rural medicine, which would strengthen the skills of medical students in tackling rural illnesses, is needed for a country where the majority of the population resides in rural areas. Further, a human resource policy for medical doctors at the national and state levels is needed, which should ensure rational staffing along with adequate remuneration based upon the area of posting. Improving facilities and the availability of medical supplies may be resource intensive; however, it is a long-term investment that may yield positive results, especially when accompanied by additional professional support. The feeling of unavailability of resources among students also suggests their reluctance to face rural realities and a disjuncture between students and rural society.

The financial factors, such as low salary and insufficient incentives, under the theme ‘g*ap between financial rewards and social disadvantages’* also emanated to be a deterrent for students’ willingness to work in rural areas. Similar findings were observed in other studies globally [15,16,37–39]. Rural practice may be encourage through direct financial incentives. However, its impact on retention of doctors from developing countries is not conclusive with the exceptions of few countries such as Mali, Zambia, and South Africa [43–49]. In developing nations, it is widelydocumented that the financial incentives must be supplemented by adequate living conditions like providing free housing and transport, communication sources (telephones, internet), jobs for spouses and recreational activities [37,38,40,42,43]. Thus inadequate financial incentives may not be the only important factor in the medical students’ decision to work in remote or rural areas. Moreover, this type of intervention can be very costly, and may not be sustainable, with a risk of the spillover effect of raising salary in other service sectors as well.

The theme also encompasses the findings of medical students’ concerns of being isolated from family, friends and other social support networks at rural posts, which are also supported by other studies [38,39,41]. Similar to the results of current study, other studies have also documented the unattractive rural lifestyle as a key factor that negatively affects the attitudes of medical students to work in rural settings [23–24, 50–51]. Organized events and informal opportunities to socialize with professional peers and local communities had a positive influence on perceptions of appreciation and recognition [42–46]. Similar to few countries that have undertaken special efforts to assist rural doctors’ spouses in finding employment in the same area, and in facilitating admission of their children to high-quality schools with boarding arrangements, other countries can plan along the similar lines. [47–49]. Some governments have implemented rotation policies, in order to limit the time spent in a difficult area within the entire professional career [11–12,17].

Very few encouraging factors emerged from the study, which influenced students’ interest to work in rural areas. They were mostly categorized under the theme-*willingness to give back to disadvantaged communities*, which contained factors like serving the poor community and desire to give back to the home community. These factors were also the dominant factors in the studies conducted globally [18–19, 21–22]. In India, the feeling of serving the poor and underprivileged is a value embedded in medical students for the inner satisfaction of their humanitarian motives [18–19].

The factors that are grouped under the theme of *broader clinical exposure* were ‘gain of better clinical experience’ and ‘the ability to easily get into post-graduation courses after serving a few years in rural service’. Participants believed that the experience of working in a rural area would be valuable and would positively influence their future career. These findings were in coherence with the findings of many other studies [39,40,42]. The societal factors that provided a boost to their motivation and self-esteem were aggregated under the broader theme of *higher status and respect*. Other studies have also quoted ‘prestige’ and ‘respect’ as the major facilitators for working in rural areas. [44–53]. It indicates that teaching students to build harmonious relationships with other individuals and groups in the villages can go a long way. It will also make communication easier and more effective.

There are various strengths of the study. Firstly, the current qualitative study has explored the discouraging and encouraging factors from three large states of India. Secondly, the themes generated out of the present exploratory study are in support with the themes emanated from the quantitative development and validation study conducted by authors, hence strengthening the findings [35]. Thirdly, COREQ guidelines for reporting the qualitative outcomes were strongly adhered to while reporting the results [34].

The study also has limitations. Firstly, it explained the opinion of medical students, which may not be truly reflecting their decision to work in rural settings. The establishment of any possible causal link between motivation and decision to work in rural settings was beyond the scope of present research. Secondly, the FGD may have led to socially desirable responses in the form of positive self-description rather than their honest opinions. Moreover, since the students have limited exposure and experience of working in rural areas, they were restricted in their ability to put together the discouraging and encouraging factors. Their judgments might also have been influenced by the hearsay from their senior counterparts. Also, improving the curriculum design of medical schools based on rural needs and requirements was beyond the scope of the study.

The findings of the present study encapsulate the important aspects that may be required to recruit and retain future doctors in rural areas. The outcome shall be helpful to policymakers and medical educators for making concerted efforts towards facilitating medical students’ decision in favor of working in rural areas. Thisendeavor will, in turn, improve the workforce in rural settings and strengthen the health systems for achieving universal health coverage. We propose that further research is necessary for different cross-cultural settings, also among medical doctors practicing in rural areas, to validate the findings of the present study.

## Conclusion

This study throws light upon the significant topic of medical students’ interest to work in rural settings and highlights discouraging and encouraging factors that deter or attract them to work in rural areas. The discouraging factors prevailed over encouraging factors which need to be identified and rectified to attract the medical students to work in rural areas. With the present state of affairs where recruitment and retention of students in rural areas appear to remain a continuous challenge for the governments across the globe, the present study offers important clues to policymakers for designing tailor-made, field-tested and cost-effective measures for attracting and retaining a workforce in rural areas. Countries like India need to provide basic infrastructure of health services in rural areas to attract and retain doctors.

Furthermore, newer approaches like community-based medical education and a longer clinical apprenticeship in rural health facilities can increase students’ exposure to the needs of rural communities leading to better community engagement. A medical curriculum review that prioritizes the health needs of the whole country is necessary, which includes early and long-term clinical exposure in rural areas during medical training, providing equitable provision of health services across urban cities and rural villages, and investing in infrastructure and basic facilities for doctors working in rural areas. The preferential selection of recruiting students from rural areas in medical studies followed by their placements in rural areas (preferentially in their native villages) might also be useful. They can in-turn become the advocates for further improvements in rural facilities. Further, reorganizing the workforce through delegation of specific tasks to less specialized health workers (task shifting) who are more willing to practice in rural area scan be a more efficient strategy towards retaining health workforce in rural areas. The program implementers and medical educators worldwide could benefit from the exchange of best practices, and learn from the design and implementation of similar strategies across various countries.
